# Lateral response artifact correction method using image stitching technique in radiochromic film dosimetry

**DOI:** 10.1002/acm2.14373

**Published:** 2024-05-02

**Authors:** Hideharu Miura, Masanori Miyazawa, Shuichi Ozawa, Tsubasa Enosaki, Masayuki Kagemoto

**Affiliations:** ^1^ Hiroshima High‐Precision Radiotherapy Cancer Center Hiroshima Japan; ^2^ Department of Radiation Oncology Institute of Biomedical & Health Sciences Hiroshima University Hiroshima Japan; ^3^ R‐TECH.INC Tokyo Japan

**Keywords:** film dosimetry, lateral response artifact, image‐stitching technique

## Abstract

**Purpose:**

Lateral response artifact (LRA) is caused by the interaction between film and flatbed scanner in the direction perpendicular to the scanning direction. This can significantly affect the accuracy of patient‐specific quality assurance (QA) in cases involving large irradiation fields. We hypothesized that by utilizing the central area of the flatbed scanner, where the magnitude of LRA is relatively small, the LRA could be mitigated effectively. This study proposes a practical solution using the image‐stitching technique to correct LRA for patient‐specific QA involving large irradiation fields.

**Methods:**

Gafchromic™ EBT4 film and Epson Expression ES‐G11000 flatbed scanner were used in this study. The image‐stitching algorithm requires a spot between adjacent images to combine them. The film was scanned at three locations on a flatbed scanner, and these images were combined using the image‐stitching technique. The combined film dose was then calculated and compared with the treatment planning system (TPS)‐calculated dose using gamma analysis (3%/2 mm). Our proposed LRA correction was applied to several films exposed to 18 × 18 cm^2^ open fields at doses of 200, 400, and 600 cGy, as well as to four clinical Volumetric Modulated Arc Therapy (VMAT) treatment plans involving large fields.

**Results:**

For doses of 200, 400, and 600 cGy, the gamma analysis values with and without LRA corrections were 95.7% versus 67.8%, 95.5% versus 66.2%, and 91.8% versus 35.9%, respectively. For the clinical VMAT treatment plan, the average pass rate ± standard deviation in gamma analysis was 94.1% ± 0.4% with LRA corrections and 72.5% ± 1.5% without LRA corrections.

**Conclusions:**

The effectiveness of our proposed LRA correction using the image‐stitching technique was demonstrated to significantly improve the accuracy of patient‐specific QA for VMAT treatment plans involving large irradiation fields.

## 1 INTRODUCTION

Radiochromic films have been widely used for patient‐specific quality assurance (QA) in various radiation therapy techniques, including three‐dimensional (3D) conformal radiation therapy, intensity‐modulated radiotherapy (IMRT), volumetric modulated arc therapy (VMAT), stereotactic radiotherapy, and proton therapy. While 2D and 3D detector array devices have improved time efficiency and volumetric evaluation, radiochromic films are advantageous in terms of high spatial resolution and are preferred as non‐active detectors in magnetic resonance imaging‐linac systems.^1^, ^2^, ^3^

The LRA in EBT film images from a flatbed scanner is a systematic error that can affect the accuracy of dose measurements. Previous studies have demonstrated that the magnitude of LRA depends on the type of film and the position of the film on the flatbed scanner as well as the irradiated dose.^4^, ^5^, ^6^, ^7^, ^8^, ^9^, ^10^, ^11^, ^12^, ^13^, ^14^, ^15^, ^16^, ^17^, ^18^ The LRA causes an increase in optical density values as the lateral distance increases from the center of the flatbed scanner. The magnitude of the LRA in the direction perpendicular to the scanning direction is relatively small at low doses and positions within approximately 5−7 cm from the center of the scanner. Furthermore, it varies depending on the type of film and flatbed scanner used.^17^, ^18^ Although the new‐generation Gafchromic EBT4 film shows improvement in reducing LRA compared to the EBT3 film, the need for LRA correction in large irradiation fields still exists.^8^ A relative correction factor is required to reduce the magnitude of the LRA.^10^, ^11^, ^12^, ^13^, ^14^, ^15^, ^16^, ^17^, ^18^ Specifically, LRA correction is crucial for avoiding erroneous measurement data for large targets, such as the head, neck, and pelvic regions. Numerous researchers have proposed methods to correct the LRA and improve the accuracy of film dosimetry.^10^, ^11^, ^12^, ^13^, ^14^, ^15^, ^16^, ^17^, ^18^ We hypothesized that the LRA could be mitigated by utilizing the central area of the flatbed scanner, as the magnitude of the LRA is relatively small near the center position of the flatbed scanner. The image‐stitching technique was employed to extract only the necessary area by combining two or more images with different viewpoints and times to generate a panoramic image.

The objective of this study is to correct the magnitude of LRA using the image‐stitching technique. The LRA is corrected by stitching together scanned films that have been shifted on the flatbed scanner.

## 2 MATERIAL AND METHODS

### 2.1 Dose response curve

The film handling procedures followed the guidelines outlined in the Task Group 235 report of the American Association of Physicists in Medicine.^19^ A sheet of EBT4 film from Lot #07052202 (20.3×25.4 cm^2^) was cut to obtain 12 pieces of 4 × 4 cm^2^ film for a dose‐response curve. A piece of film was placed on the central axis (CAX) in a water‐equivalent phantom (Kyoto Kagaku Co., Ltd, Kyoto, Japan) with 10 cm of buildup material above and below the film. The source‐to‐film distance was set at 100 cm. Twelve pieces of film were irradiated at doses ranging from 25 to 1000 cGy using a field size of 10 × 10 cm^2^ with a 6 MV beam produced by a Varian TrueBeam STx linear accelerator (Varian Medical Systems, Palo Alto, CA). One piece of the film was left unirradiated to obtain background readings. Prior to any film irradiation, linac calibration was verified using a Farmer‐type ionization chamber (Model N30013; PTW, Freiburg, Germany) connected to a RAMTEC electrometer (Toyo Medic, Tokyo, Japan).

All irradiated films were scanned approximately 24 h after irradiation to minimize the effects of post‐exposure darkening. An Epson Expression ES‐G11000 (Epson Seiko Corporation, Nagano, Japan) document flatbed scanner and EPSON Scan v3.49 software were used in this study. A clear glass compression plate was placed on the film to mitigate natural curling.^16^, ^20^ Films were placed at the center of the flatbed scanner in portrait orientation. Portrait orientation indicates that the long dimension of the original sheet is parallel to the scan direction. Films were scanned in professional mode with image adjustments and color correction turned off, using a resolution of 75 dots per inch in the red color channel. Data were saved in an uncompressed tagged image file format. For the scanned images, a region of interest (ROI) of 50 × 50 pixels was set for each film piece, and the median pixel value in the ROI was measured to create the dose‐response curve. Only the red channel was used in this study because the LRA of the red color channel is most prominent.^16^

### 2.2 Lateral response artifact

The films (20.3×25.4 cm^2^) were cut into rectangular pieces measuring 20.3×4 cm^2^ and irradiated to investigate the magnitude of LRA in the portrait orientation. Each film was irradiated at doses of 100, 200, 300, 400, 500, and 600 cGy in a water‐equivalent phantom with 10 cm of buildup material above and below the film. An 18×18 cm^2^ open field with 6 MV photons was exposed at the center of the film. These films were scanned at the center of the flatbed scanner, aligning the short dimension of the cut piece of film parallel to the scan direction. Dose profiles on central axis of the beam in the lateral direction were then acquired and compared to the treatment planning system (TPS)‐calculated doses from the Eclipse TPS (Varian Medical Systems, Palo Alto, CA, USA) using the Acuros XB algorithm.

### 2.3 Image stitching

**FIGURE 1:**
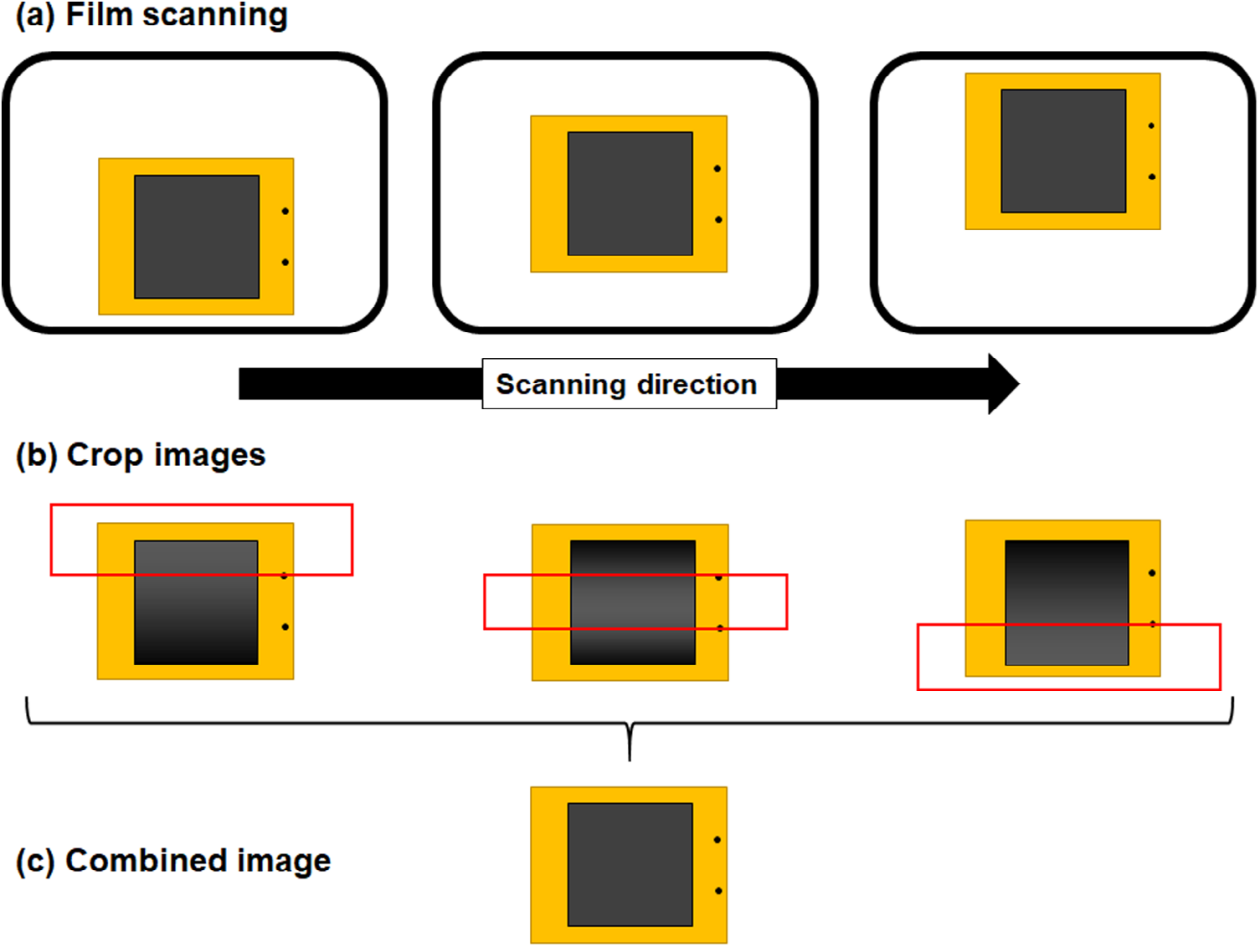
Illustration of the film scanning and image‐stitching procedures: (a) Each film was scanned in three positions on the scanner to acquire data for the upper, middle, and lower areas, respectively. (b) Cropped areas (indicated by red squares) were obtained from these images to generate three images of the necessary areas. (c) The final combined image was then produced.

Two spots were marked on a full sheet of film using a permanent black marker pen to determine the positions for image stitching. Each spot has an approximate diameter of 1 mm. Since the marker values and the measurements on the film at high doses could be relatively similar, we determined the marker position and the size of the search area of interest in advance. The spots were marked at approximately 7 and 14 cm along the short dimension of a full sheet of film, with the scanning direction of the spot approximately 1 cm from the film edge for each. Our proposed LRA correction requires three scans at different positions on the flatbed scanner for each film to combine the images. A paper ruler was used to accurately align the film on the flatbed scanner. Initially, film was placed on the lower part of the flatbed scanner and scanned for the upper image. For the middle and lower images, the film was scanned by sliding it approximately 7 and 14 cm, respectively, as shown in Figure 1a. Figure 1b illustrates the image‐stitching process for combining each image, and the scanned data that were relatively central to the flatbed scanner were used to create the combined data. The algorithm utilized in this study identifies the maximum value within the search area to precisely determine the marker locations. The images were cropped horizontally based on the detected markers. Finally, Figure 1c shows the combined image for the proposed LRA correction. To combine the images, the films that were scanned in the lower, middle, and upper positions on the scanner were used for the upper, middle, and lower image areas.

### 2.4 Verification

To test the effectiveness of our proposed LRA correction, we measured a field size of 18 × 18 cm^2^ and four VMAT treatment plans using EBT4 films, and compared them with the TPS‐calculated doses. For this purpose, we utilized 20.3 × 25.4 cm^2^ sheets of film. We verified three dose levels for the 18 × 18 cm^2^ open field: 200, 400, and 600 cGy. These levels encompassed conventional treatment plan doses, as well as both realistic and unrealistic doses involving large irradiation fields. The phantom setup conditions for the 18 × 18 cm^2^ open field are the same as those described in Section 2.2. In terms of phantom mapping, we employed head and neck VMAT treatment plans with a dose of 200 cGy. These plans were designed using the Eclipse TPS with 6 MV photons. A calculation grid size of 2 mm and the Acuros XB algorithm were used for dose calculation. The film was placed at the isocenter and sandwiched between two 5 cm water‐equivalent phantoms. The films were scanned at different flatbed scanner positions and then combined for LRA correction, as described in Section 2.3. The combined film was then calculated and compared with the TPS‐calculated dose using the gamma analysis method in DD‐IMRT ver. 18.3 (R‐TECH.INC., Tokyo, Japan), applying a criterion of 3% dose difference and a 2 mm distance‐to‐agreement with a 30% threshold.

## 3 RESULTS

**FIGURE 2:**
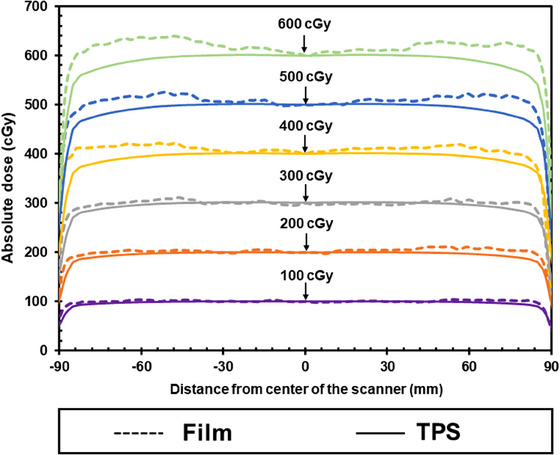
Lateral dose profiles acquired using EBT4 films at various dose levels with an 18 × 18 cm^2^ open field. The absolute dose was calculated using a treatment planning system (TPS) for comparison with the film measurements.

Figure 2 shows the transverse dose profiles in the lateral direction of the EBT4 films irradiated with an 18 × 18 cm^2^ open field and the TPS‐calculated dose. The data represent films exposed to doses of 100, 200, 300, 400, 500, and 600 cGy. The film measurements demonstrated a tendency to be higher as the position approached the lateral edge of the flatbed scanner. This trend is influenced by both the film's position on the flatbed scanner and the dose level, with smaller deviations at low doses and larger deviations at higher doses. At a lateral distance of approximately 3.5 cm, the film measurements were within approximately 3% of the TPS calculations. However, at lateral positions of approximately 8 cm, the EBT4 film exhibited overresponses of 7.7%, 7.6%, 5.6%, 5.0%, 8.2%, and 9.0% for doses of 100, 200, 300, 400, 500, and 600 cGy, respectively.

**FIGURE 3:**
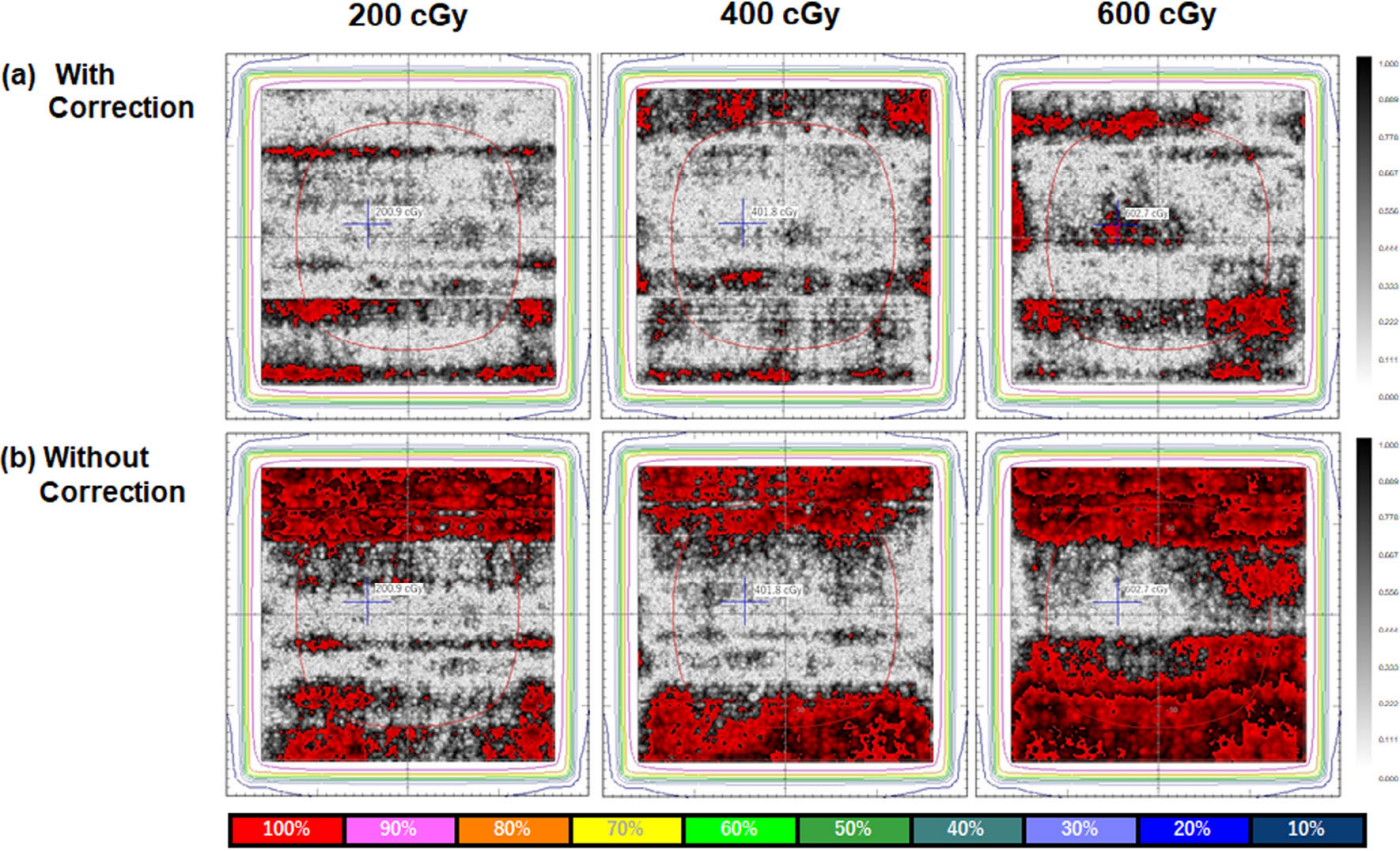
The gamma distributions were analyzed (a) with or (b) without LRA corrections, using doses of 200, 400, and 600 cGy with 18 × 18 cm^2^ open fields. The gray color indicates the gamma distribution resulting from the film measurements, while the red color indicates locations where the gamma values (3%/2 mm) calculated between the film and treatment planning system (TPS)‐calculated dose exceed 1.

**FIGURE 4:**
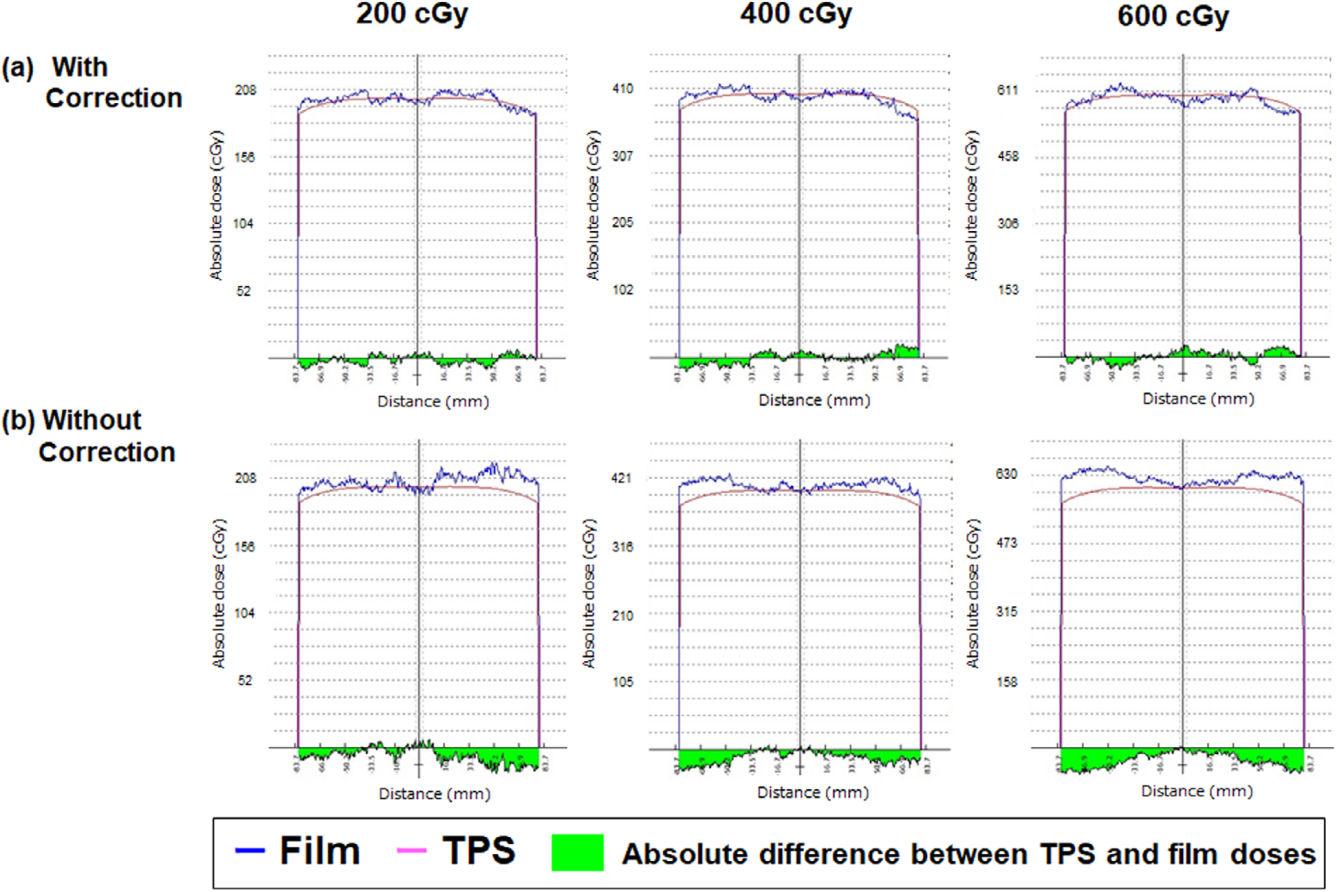
Dose profiles were acquired (a) with or (b) without LRA corrections, using doses of 200, 400, and 600 cGy with 18 × 18 cm^2^ open fields. The dose profiles were acquired perpendicular to the scan direction, and the absolute dose was calculated using the treatment planning system (TPS) for comparison with the film measurements.

Figures 3 and 4 present the results of the gamma analysis and central dose profile data obtained from the measured dose films and the TPS‐calculated dose using an 18 cm × 18 cm^2^ open field, with and without LRA corrections. Film measurements without LRA correction showed higher dose curves in the off‐center positions of the flatbed scanner, with a maximum deviation of approximately 9.5%, 8.0%, and 8.6% for doses of 200, 400, and 600 cGy, respectively, compared to the TPS‐calculated doses. However, the corrected film values were in good agreement with the TPS‐calculated doses, showing improved results for the three dose levels, despite variations in the magnitude of the LRA. For doses of 200, 400, and 600 cGy, the gamma passing rates with and without LRA corrections were 95.7% versus 67.8%, 95.5% versus 66.2%, and 91.8% versus 35.9%, respectively. An image stitching artifact was found at the border where the individual EBT film images were stitched together.

**FIGURE 5:**
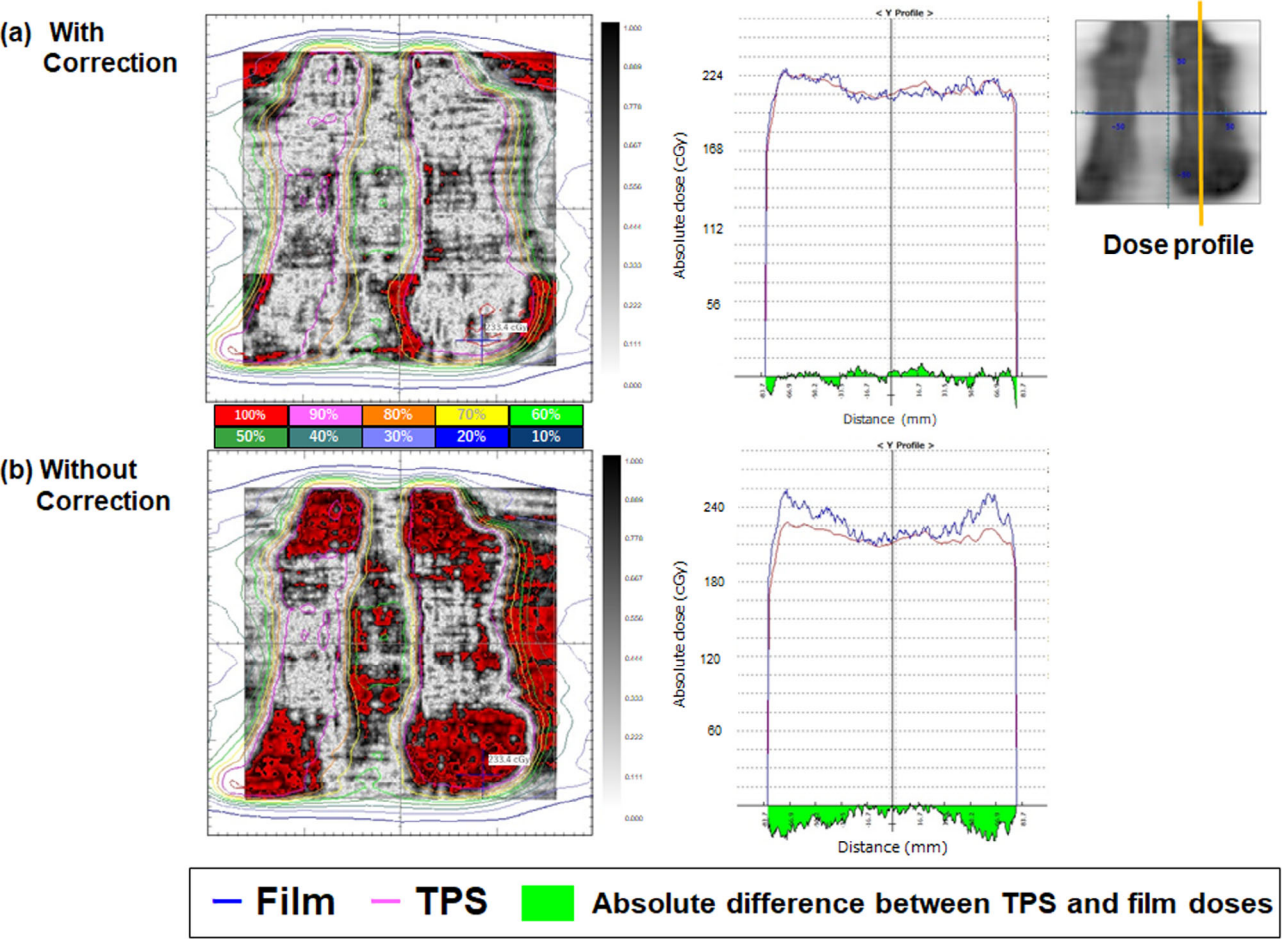
The gamma distributions of the clinical VMAT treatment plan were analyzed (a) with or (b) without LRA corrections. The gray color indicates the gamma distribution resulting from the film measurement, while the red color indicates locations where the gamma values (3%/2 mm) calculated between the film and treatment planning system (TPS)‐calculated dose exceed 1. The displayed dose profiles were measured along the yellow line in the upper‐right figure.

Figure 5 presents a comparison of the gamma analysis and dose profile of the clinical VMAT treatment plan with and without LRA correction. Table 1 lists the gamma passing rates of the clinical VMAT treatment plan with and without LRA correction. The average ± standard deviation in gamma analysis for the clinical VMAT treatment plans with LRA corrections was 94.1% ± 0.4%, and without LRA corrections was 72.5% ± 1.5%. This indicates that our proposed LRA correction demonstrates an improvement in mitigating the magnitude of LRA compared to using no LRA correction film in the clinical VMAT treatment plan. Additionally, the dose profiles of the dose maps in Figures 4 and 5 present an improvement in the magnitude of LRA across the exposed areas with large irradiation fields.

**TABLE 1:** Comparison of pass rates for gamma analysis with and without LRA correction.

	With LRA correction (%)	Without LRA correction (%)
Plan 1	94.4	73.3
Plan 2	93.5	72.4
Plan 3	93.8	70.2
Plan 4	94.6	74.1

Abbreviation: LRA, lateral response artifact.

## 4 DISCUSSION

We propose a method to correct the LRA of radiochromic films scanned image using the image‐stitching technique. First, we assessed the magnitude of LRA on the ES‐G11000 flatbed scanner by examining the film measurements at various irradiation doses. At an off‐center distance of approximately 3.5 cm, all film measurements showed a relative deviation of less than ± 3% compared to the TPS‐calculated dose without LRA correction. We investigated the magnitude of LRA up to a dose of 6 Gy. Doses larger than 6 Gy may exhibit a greater magnitude of LRA, and our proposed LRA correction might be insufficient for these higher doses. In general, conventional IMRT or VMAT treatments with large fields typically deliver up to a dose of approximately 5 Gy. Therefore, our measurement up to 6 Gy is considered sufficient to support the magnitude of LRA involving clinically large irradiation fields. As the dose delivered to the film increased, so did the LRA at the off‐center on the flatbed scanner. Previous studies have reported that the magnitude of the LRA varies as a function of dose and lateral scan position depending on the type of film and flatbed scanner used.^4^, ^5^, ^6^, ^7^, ^8^, ^9^, ^10^, ^11^, ^12^, ^13^, ^14^, ^15^, ^16^, ^17^, ^18^ LRA comes from differences in optical path length inside the film with increasing distance from the center of the scanner. It also depends on how the scanner's optical mirror system responds to light polarization from the film.^6^ Based on our results of the magnitudes of the LRA on the ES‐G11000 flatbed scanner, it was necessary to correct the LRA for a large irradiation field.

Second, we utilized an image‐stitching technique on a relatively small area to combine the images for LRA correction regarding the magnitude of LRA. In case of large irradiation field sizes without LRA correction, most failure points were located near the edges of the fields. However, after applying our proposed LRA correction, the magnitude of LRA was significantly reduced, as shown in Figures 3, 4, 5. Comparing the results with and without LRA correction highlights the effectiveness of our proposed LRA correction, resulting in an improvement of the gamma passing rate by 27.9%, 29.3%, and 55.9% at doses of 200, 400, and 600 cGy, respectively, using 3%/2 mm gamma criteria with a 30% threshold. Furthermore, our proposed LRA correction exhibited an average 21.6% improvement in the gamma passing rate for the clinical VMAT treatment plan, using 3%/2 mm gamma criteria with a 30% threshold. Several researchers have reported the applicability of the LRA correction technique using dual‐ or triple‐channel corrections with calibration data acquired from different film positions in the lateral direction.^10^, ^12^, ^15^, ^16^, ^17^, ^18^ A correction factor must be applied to the dose measurements to account for the LRA. To assess the magnitude of LRA and calculate the correction factor, it is necessary to scan a number of films irradiated at different doses and positions on the flatbed scanner in the lateral direction. This step is crucial because scanners for film measurement exhibit dose‐dependent LRA patterns. Our proposed LRA correction method can address the nonuniformity of scanned images of radiochromic films without requiring the correction matrices over the entire scan field. It provides an improvement for patient‐specific QA using simple methods. However, our proposed LRA correction requires three scans for each film to combine the images. Previous authors have reported differences in color‐dependent sensitivity and lateral non‐uniformity between Light Emitting Diode (LED)‐type flatbed scanners (Epson V800) and cold cathode fluorescent lamp‐type scanners (Epson 11000XL).^9^ Regarding the un‐irradiated film, the center‐to‐off‐axis ratio for the V800 and 11000XL scanners is approximately 0.960 and 0.995, respectively. The LED‐type flatbed scanner (Epson V800) exhibits a significant impact from lateral non‐uniformity, and our proposed LRA correction may not be applicable with only three shifted images. To apply our proposed LRA correction method to other scanners, it might be necessary to divide the scanning area into smaller areas with more frequent sliding.

A limitation of this study was that only one scanner was used (ES‐G11000). The film was scanned by sliding it approximately 7 cm, and the resulting images were combined using the image‐stitching technique. The ES‐G11000 flatbed scanner had a relatively small scanning area, with a range suitable for this method of approximately ± 3.5 cm. This feature provided a reasonable balance between the number of scans and the irradiated dose. In our study, we used only one single lot of EBT4 film with a single flatbed scanner for dosimetry using the red color channel. In a previous study, four different film lots and four different flatbed scanners were used to validate the correction method across all three color channels for various doses up to 1600 cGy.^16^ They reported that compensating for the LRA was independent of the production lot of the EBT3 film. Our proposed LRA correction for a specific scanner may be applicable to other lots numbers and scanner types, and to dosimetry using the other color channels. However, further confirmation is needed of its wider applicability. It is important to note that the magnitude of LRA depends on the type of scanner used, type of film, position of the film on the flatbed scanner as well as the irradiated dose.^4^, ^5^, ^6^, ^7^, ^8^, ^9^, ^10^, ^11^, ^12^, ^13^, ^14^, ^15^, ^16^, ^17^, ^18^ The feathering or alpha blending approach could minimize seam artifacts by smoothing the transition between the images. However, our proposed method did not implement these approaches because we used simple techniques for image stitching.^21^

## 5 CONCLUSIONS

We have proposed a process that utilizes the image‐stitching technique to correct the LRA. In our proposed LRA correction method, correction factors are not necessary, but three scans at different positions on the flatbed scanner for each film are required to combine the images. The effectiveness of our proposed LRA correction is demonstrated by a significant improvement in the accuracy of patient‐specific VMAT treatment plans involving large irradiation fields.

## AUTHOR CONTRIBUTIONS

All authors contributed substantially to the design of the study. Hideharu Miura conducted the study. Masanori Miyazawa developed the software. All authors contributed to the analysis and interpretation of the results. Hideharu Miura drafted the manuscript and all authors provided critical feedback on revising the manuscript for important intellectual content and all authors gave their final approval of the version to be published.

## CONFLICT OF INTEREST STATEMENT

Masanori Miyazawa is an employee of R‐TECH.INC.
